# Bruton Tyrosine Kinase Inhibitors in Immune-Mediated Skin Diseases: Current Evidence and Future Directions

**DOI:** 10.3390/ijms27209375

**Published:** 2026-10-21

**Authors:** Zihan Wang, Junchen He, Ting Zhang, Jiayu Peng, Lulu Xie, Bingrun Li, Cunhao Shan, Litao Zhang, Zuotao Zhao

**Affiliations:** 1 Institute of Dermatology, Tianjin Academy of Traditional Chinese Medicine, Tianjin 300120, China; zh_wang1001@163.com (Z.W.); hjc420359745@gmail.com (J.H.); tingzhang1113@163.com (T.Z.); 18744019367@163.com (J.P.); 1120210682@mail.nankai.edu.cn (L.X.); zhanglitao@medmail.com.cn (L.Z.); 2 Graduate School, Tianjin University of Traditional Chinese Medicine, Tianjin 301617, China; libr0011@126.com (B.L.); xianrujid48963@163.com (C.S.)

**Keywords:** BTK inhibitors, immune-mediated skin diseases, chronic spontaneous urticaria, pemphigus

## Abstract

Bruton tyrosine kinase (BTK) is a central signaling molecule that links the B-cell receptor (BCR), Fc receptors, and Toll-like receptors (TLRs). By coordinating signaling in B cells, mast cells, macrophages, and other immune cells, BTK occupies an important position in cutaneous immune responses. BTK inhibitors (BTKis) can inhibit B-cell and Fc-receptor-dependent effector-cell signaling, providing a mechanistic rationale for several immune-mediated skin diseases. However, these cellular effects should not be equated with suppression of established autoantibody production or durable disease modification. This review summarizes the biological functions of BTK and critically evaluates clinical and translational evidence in chronic spontaneous urticaria (CSU), atopic dermatitis (AD), pemphigus, psoriasis, and hidradenitis suppurativa (HS). Evidence is strongest for short-term symptom control in CSU cases. Clinical value remains uncertain in pemphigus and HS, rilzabrutinib did not meet its primary AD efficacy endpoint and topical atuzabrutinib showed no exploratory efficacy benefit, and psoriasis evidence is preclinical. Priorities include prospective validation of predictive biomarkers, disease-specific confirmatory trials, agent- and setting-specific safety assessments, and long-term and real-world follow-up.

## 1. Introduction

Bruton tyrosine kinase (BTK) is a non-receptor tyrosine kinase in the Tec family and a core component of B-cell receptor (BCR) signaling [1,2]. It contributes to B-cell development and BCR-dependent activation [3,4]. Antigen engagement of the BCR triggers signaling cascades that regulate B-cell activation and differentiation [4,5]. BTK is also expressed in monocytes and macrophages, dendritic cells, basophils, and mast cells. In these cells, it contributes to Toll-like receptor (TLR) and Fc receptor-mediated inflammatory signaling. BTK therefore links adaptive and innate immune responses [2,6,7].

A growing body of evidence associates dysregulated BTK signaling with several immune-mediated diseases [8,9,10]. The relative contributions of B-cell and myeloid-cell pathways vary across these conditions. BTK participates in signaling downstream of B-cell receptors in B cells and Fc receptors, including FcεRI, in myeloid cells [11]. These mechanistic effects alone should not be interpreted as evidence of sustained suppression of pathogenic antibodies in patients. Clinical evidence must therefore distinguish rapid effects on effector cells from effects on established autoantibody production and long-term disease course.

Immune-mediated skin diseases include chronic spontaneous urticaria (CSU), atopic dermatitis (AD), and pemphigus. CSU is characterized by recurrent wheals and/or angioedema [12]. AD is a chronic inflammatory skin disease that can substantially impair quality of life [13], whereas pemphigus comprises autoimmune blistering diseases affecting the skin and mucous membranes [14]. The distinct pathogenic mechanisms of these conditions underscore the need for disease-specific evaluation of emerging targeted therapies. BTKi studies have produced different levels of evidence across this spectrum. Phase III trials support symptom improvement in CSU cases [15], whereas early clinical signals in individuals with pemphigus were not confirmed by the primary endpoint of the phase III PEGASUS trial [16,17]. In AD cases, rilzabrutinib did not meet its primary efficacy endpoint and topical atuzabrutinib showed no exploratory efficacy benefit; HS findings are preliminary, and the psoriasis studies reviewed here are preclinical [18,19,20,21,22,23].

Against this background, this review has two aims. First, it summarizes the central biological functions of BTK in cutaneous immunity and its role across interconnected immune-cell networks. Second, it critically evaluates clinical and translational evidence for BTKis in CSU, AD, pemphigus, psoriasis, and hidradenitis suppurativa (HS). Particular attention is given to the distinction between mechanistic rationale, preclinical findings, exploratory clinical signals, and confirmed clinical efficacy. This evidence hierarchy is necessary to define what is established, what remains uncertain, and which hypotheses require prospective validation.

## 2. Biological Basis of BTK in Cutaneous Immunity

**Figure 1:**
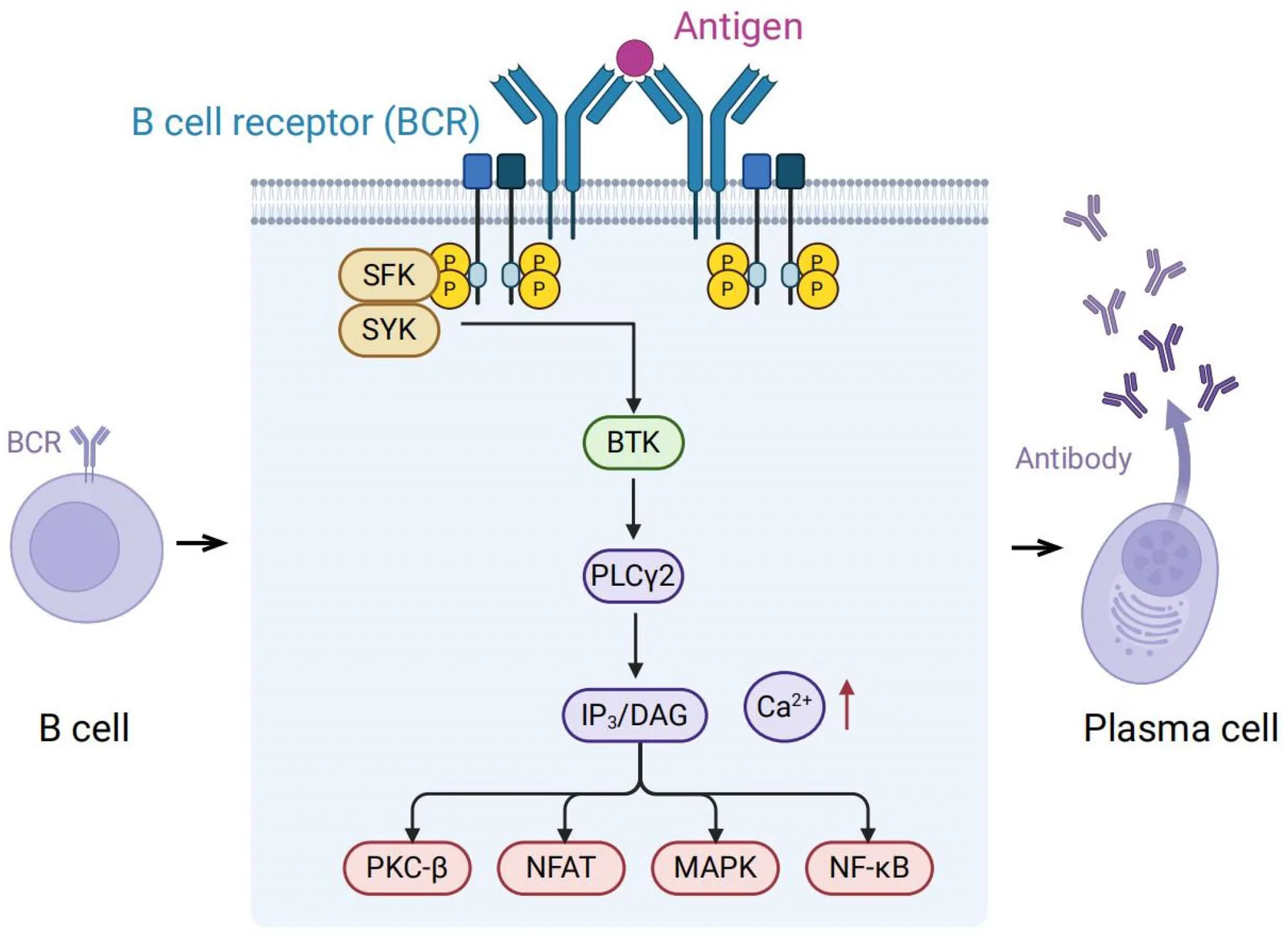
B-cell receptor-dependent BTK signaling. Antigen engagement of the B-cell receptor (BCR) activates Src-family kinases (SFKs) and spleen tyrosine kinase (SYK), followed by activation of Bruton tyrosine kinase (BTK) and phospholipase Cγ2 (PLCγ2). PLCγ2 generates inositol 1,4,5-trisphosphate (IP3) and diacylglycerol (DAG), increases intracellular Ca2+, and activates protein kinase C beta (PKC-β), nuclear factor of activated T cells (NFATs), mitogen-activated protein kinase (MAPK), and nuclear factor kappa B (NF-κB), thereby promoting B-cell activation and differentiation toward antibody-secreting cells. Black arrows indicate signaling activation; circled *p* indicates phosphorylation. Created with BioRender.com.

Agammaglobulinemia was described by Bruton in 1952 [24]. The gene disrupted in X-linked agammaglobulinemia was identified in 1993 [25]. BTK is classified within the Tec family of non-receptor tyrosine kinases [1,2]. Figure 1 summarizes its role in BCR signaling and B-cell activation [1,2,4,5].

BTK is also widely expressed in innate immune cells, including monocytes and macrophages, dendritic cells, basophils, and mast cells. In these compartments, BTK interacts with signaling initiated by chemokine receptors, TLRs, and Fc receptors. It participates in FcγR-, FcεRI-, and TLR-associated inflammatory signaling, including Fc-receptor-dependent effector-cell activation [2,6,7]. This distribution explains why BTK functions not only in B-cell-mediated adaptive immunity but also as an important regulator of immune-cell-mediated inflammation [1,6]. Its position across several cell types permits a single molecular intervention to affect antibody generation, immune-complex responses, myeloid activation, and immediate mediator release.

BTK signaling regulates B-cell activation and Fc-receptor-dependent effector-cell responses, providing a mechanistic rationale for inhibition across immune-mediated diseases [2,11,26]. Binding mode and selectivity differ among BTK inhibitors; agents under investigation include both covalent and non-covalent inhibitors [2,26]. These pharmacological differences require agent-specific assessment of efficacy and safety [7,11].

In summary, BTK occupies a central position in B-cell development, intracellular signaling, and cross-cellular immune regulation. Precise regulation of this pathway helps maintain immune homeostasis, whereas dysregulation can shape the pathological course of several immune-mediated skin diseases. A detailed understanding of BTK-dependent mechanisms and interactions among immune-cell populations is therefore essential for selecting appropriate clinical settings, interpreting variable responses, and developing individualized BTKi treatment strategies.

## 3. Clinical Development of BTK Inhibitors in Immune-Mediated Skin Diseases

### 3.1. Chronic Spontaneous Urticaria

CSU is an inflammatory skin disease characterized by recurrent wheals, angioedema, or both for more than six weeks [12,27]. Its fluctuating symptoms can markedly impair sleep, daily functioning, and quality of life [12].

**Figure 2:**
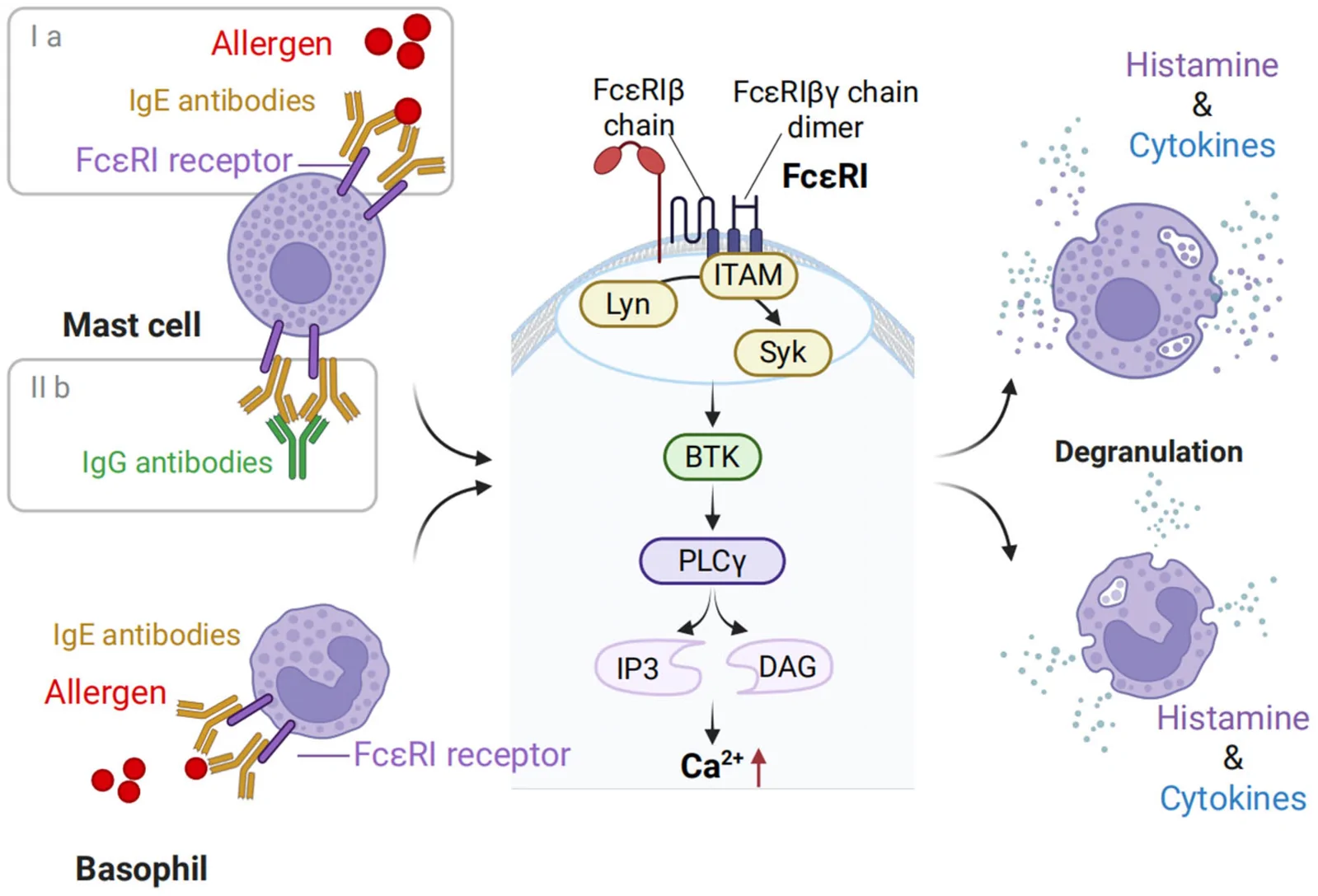
BTK-dependent FcεRI signaling in mast cells and basophils in chronic spontaneous urticaria. In type I autoimmune or allergic chronic spontaneous urticaria, allergen- or autoallergen-bound immunoglobulin E (IgE) signals through the high-affinity IgE receptor (FcεRI). In type IIb autoimmune disease, immunoglobulin G (IgG) autoantibodies directed against IgE or FcεRI can activate the same effector-cell pathway. FcεRI signaling proceeds through Lyn, immunoreceptor tyrosine-based activation motifs (ITAMs), SYK, BTK, phospholipase C gamma (PLCγ), IP3, DAG, and Ca2+ mobilization, leading to mast-cell and basophil degranulation and release of histamine and cytokines. Arrows indicate signaling activation [7,27]. Created with BioRender.com.

Type I autoimmune (autoallergic) and type IIb autoimmune mechanisms in CSU converge on mast-cell activation [12,27]. Figure 2 summarizes the BTK-dependent FcεRI signaling axis in mast cells and basophils; its clinical relevance is considered below. Supporting this mechanistic rationale, Gimeno et al. showed that remibrutinib inhibited human basophil and mast-cell activation induced in vitro by sera from patients with chronic urticaria, irrespective of their clinical response to omalizumab [28]. These findings support an effect on effector-cell activation but do not establish clinical efficacy in omalizumab non-responders.

Other BTKis, including fenebrutinib and rilzabrutinib, have produced preliminary clinical signals in CSU patients, but the evidence remains limited outside the remibrutinib program (Table 1) [29,30].

Evidence is most mature for remibrutinib, which improved CSU activity in the REMIX phase III program and was generally well-tolerated; study design and quantitative outcomes are summarized in Table 1 [15]. The US Food and Drug Administration approved remibrutinib for CSU on 30 September 2025 [31]; its approval in China was announced on 25 November 2025 [32].

### 3.2. Atopic Dermatitis

AD is a chronic inflammatory skin disease characterized by epidermal-barrier dysfunction and predominantly type 2 immune inflammation [13,33]. Increased IgE concentrations and severe pruritus are common [13]. Fc-receptor signaling provides a mechanistic rationale for investigating BTK inhibition, but this rationale does not establish clinical benefit in AD [11,26].

In a phase II AD trial, systemic rilzabrutinib did not meet the primary EASI endpoint or key secondary efficacy endpoints; improvements in continuous itch measures were exploratory [18]. Topical atuzabrutinib showed no benefit over vehicle in exploratory lesion and pruritus assessments in a phase IIa study whose primary objectives were safety and tolerability (Table 1) [19].

Pruritus in AD is related to neuroimmune signaling involving IL-31, IL-4, and IL-13 and mast-cell pathways [34]. The primary EASI and key secondary efficacy endpoints of the rilzabrutinib study were not met, and the atuzabrutinib study provided no exploratory efficacy signal [18,19]. These findings do not establish clinically meaningful control of AD. Combining a BTKi with an IL-4 or IL-13 pathway inhibitor such as dupilumab, or with a Janus kinase inhibitor, is therefore a speculative, hypothesis-generating proposal rather than a clinically supported strategy. The studies reviewed here do not establish the efficacy or safety of these combinations, and any prospective evaluation would need to address infection, bleeding, and cumulative immunomodulatory risk.

### 3.3. Pemphigus

Pemphigus is an autoantibody-mediated blistering disorder [14]. Its pathogenesis is closely associated with pathogenic autoantibodies against desmoglein (DSG) 1 and DSG3 [35]. BTK supports activation of autoreactive B cells and FcγR-dependent signaling in myeloid effector cells, providing a biological rationale for BTK inhibition [26]. However, this rationale does not establish that treatment will eliminate existing DSG-antibody production. Mechanistic effects on B cells and effector cells alone cannot demonstrate sustained suppression of pathogenic antibodies in patients. Changes in antibody titers, clinical remission, and glucocorticoid exposure should therefore be evaluated as distinct outcomes.

In the single-arm phase II BELIEVE study, rilzabrutinib showed a clinical and glucocorticoid-sparing signal; however, the uncontrolled design precludes the attribution of these changes to rilzabrutinib alone (Table 1) [16].

A single-arm phase II study of tirabrutinib in refractory pemphigus reported descriptive remission and glucocorticoid-reduction signals, but it cannot establish comparative efficacy or durable disease modification (Table 1) [36].

Topical BTK inhibition has also been explored preclinically. Topical PRN473 reduced IgG-mediated passive Arthus reactions in rats and IgE-mediated passive cutaneous anaphylaxis in mice, with low systemic accumulation [37]. The same report described efficacy of oral, rather than topical, PRN473 in canine pemphigus foliaceus. These findings demonstrate local pharmacological activity in animal models but do not establish efficacy in patients with pemphigus or support topical BTK inhibition as a clinical treatment strategy.

### 3.4. Hidradenitis Suppurativa

HS is a chronic, recurrent, and destructive inflammatory skin disease initiated by occlusion of the follicular infundibulum. It presents with recurrent painful nodules and abscesses in apocrine-gland-bearing areas and may progress to draining tunnels and scarring. These manifestations impose a substantial burden on quality of life [38]. Tumor necrosis factor alpha inhibition with adalimumab, IL-17A inhibition with secukinumab, and dual IL-17A/F inhibition with bimekizumab are approved for moderate-to-severe HS. Nevertheless, inadequate response or loss of response remains common, supporting investigation of additional therapeutic approaches that target complementary immune pathways [39].

Transcriptomic analysis of HS lesions showed increased expressions of BTK, SYK, JAK1, JAK3, and several STAT family members, together with inflammatory cytokines including IL-6, interferon gamma, and IL-23A [40]. JAK–STAT signaling is therefore one of several pathways under investigation in HS; JAK inhibitors have also been evaluated across inflammatory skin diseases [41]. Their clinical positioning and long-term safety require disease- and agent-specific evaluations.

BTK has attracted interest in HS studies because it links innate and adaptive immune mechanisms. A randomized phase II platform study provided an early signal for remibrutinib, but its non-monotonic dose pattern and small cohorts require confirmation; trial design and response data are summarized in Table 1 [20].

Exploratory patient-reported pain, itch, and quality-of-life outcomes have also been reported for remibrutinib in HS patients; these descriptive results come from the same platform-study cohort and remain preliminary (Table 1) [42].

Taken together, the HS findings are promising but preliminary. They justify further controlled investigation of BTK dependence, dose-response, and durability, but they do not yet support positioning BTKis within the HS treatment algorithm or comparing them with approved cytokine-directed therapies.

### 3.5. Psoriasis

**Figure 3:**
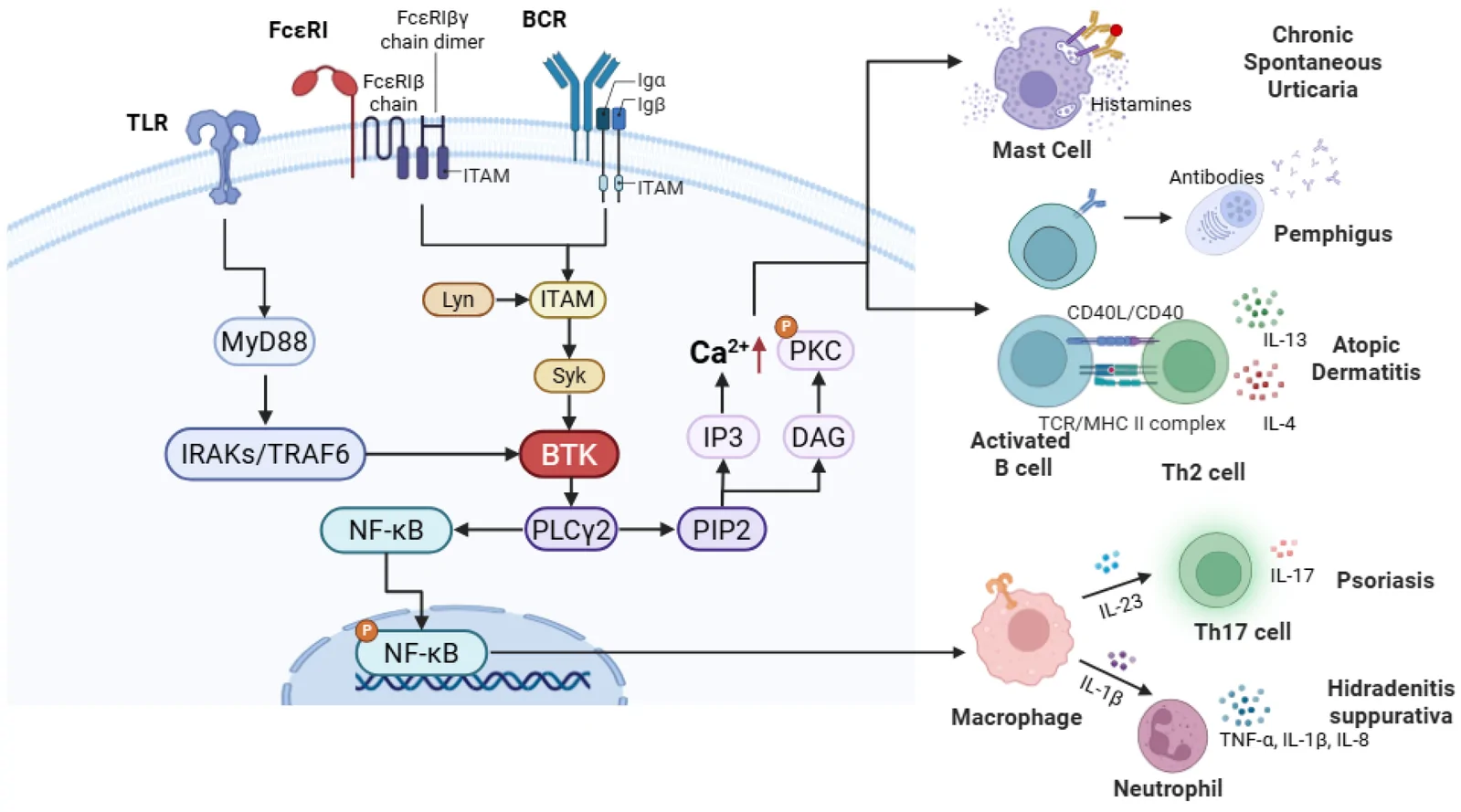
BTK-centered signaling nodes across immune-mediated skin diseases. Signals initiated by Toll-like receptors (TLRs), FcεRI, and the B-cell receptor (BCR) converge on BTK and activate nuclear factor kappa B (NF-κB) and PLCγ2-dependent Ca2+ signaling. These pathways can influence mast-cell degranulation in chronic spontaneous urticaria, B-cell and autoantibody responses in pemphigus, B-cell–T-helper type 2 (Th2) interactions in atopic dermatitis, macrophage–T-helper type 17 (Th17) signaling in psoriasis, and macrophage/neutrophil-associated inflammation in hidradenitis suppurativa. The figure summarizes candidate biological relationships; not every cell-specific connection has been established as BTK-dependent in each disease. The disease-specific evidence includes general pathway biology, tissue-expression studies, and preclinical intervention studies [2,11,21,23,26,27,40,44]. FcεRI, high-affinity IgE receptor; IRAKs, interleukin-1 receptor-associated kinases; MyD88, myeloid differentiation primary response 88; PIP2, phosphatidylinositol 4,5-bisphosphate; TCR/MHC II, T-cell receptor/major histocompatibility complex class II; TNF-α, tumor necrosis factor alpha; TRAF6, TNF receptor-associated factor 6. Arrows indicate signaling activation. Created with BioRender.com.

Psoriasis is an immune-mediated inflammatory skin disease. A global burden analysis documented an increase in psoriasis cases between 1990 and 2021 [43]. The IL-23/IL-17 axis and downstream ACT1/TRAF6/TAK1/NF-κB signaling contribute to inflammation in experimental psoriasis models [44]. Candidate BTK-dependent pathways are summarized in Figure 3, but their therapeutic relevance in patients remains unconfirmed [21,23].

BTK inhibition has shown activity in animal models of psoriasis. In an imiquimod-induced mouse model, preventive treatment with PCI-32765 reduced IL-23-associated responses in dendritic cells and IL-17A-associated responses in γδ T cells [21]. These findings provide preclinical evidence for the role of BTK-associated signaling in psoriatic inflammation.

Investigators subsequently developed a nanostructured lipid-carrier gel containing both ibrutinib and curcumin. Topical administration reduced disease-severity scores and inflammatory mediators in an imiquimod-induced mouse model [22]. Because the formulation combined a BTKi with curcumin and a delivery system, the contribution of BTK inhibition cannot be isolated. Together, psoriasis studies are preclinical and hypothesis-generating; they do not establish clinical efficacy, safety, or a place for BTKis within current psoriasis treatment strategies.

The design and principal outcomes of the available clinical studies are summarized in Table 1.

**Table 1:** Selected clinical studies of BTK inhibitors in immune-mediated skin diseases.

Study	Drug	Disease	Phase	Sample Size	Treatment Groups	Main Endpoint and Result
Metz 2021 [29] NCT03137069	Fenebrutinib	CSU	II	93	Placebo; 50 mg once daily (QD); 150 mg QD; 200 mg twice daily (BID)	Change from baseline in UAS7 at week 8; met at 150 mg QD and 200 mg BID, but not 50 mg QD
Giménez-Arnau 2025 [30] NCT05107115	Rilzabrutinib	CSU	II	160	Placebo; rilzabrutinib 400 mg QD, 400 mg BID, or 400 mg three times daily	Change from baseline in ISS7 or UAS7 at week 12; met for 1200 mg/day in the 143 omalizumab-naive patients in the primary analysis
Kaul 2021 [45] NCT03918980	Remibrutinib	Healthy participants with or without asymptomatic atopic diathesis	I	156	SAD: 0.5–600 mg; MAD: 10–600 mg QD or 100–200 mg BID; placebo	Safety and tolerability (primary); PK and PD measures; no dose-limiting toxicity in healthy participants
Maurer 2022 [46] NCT03926611	Remibrutinib	CSU	II	311	Placebo; 10, 35, or 100 mg QD; 10, 25, or 100 mg BID	Change from baseline in UAS7 at week 4 and safety; all dose groups improved versus placebo (nominal *p* < 0.0001)
Jain 2024 [47] NCT04109313	Remibrutinib	CSU	IIb	194 treated	100 mg BID	Long-term safety (primary); uncontrolled extension showed sustained UAS7 responses throughout week 52
Metz 2025 [15] NCT05030311; NCT05032157	Remibrutinib	CSU	III	925	Placebo; 25 mg BID	Change from baseline in UAS7 at week 12; met in both REMIX trials (*p* < 0.001)
Lee 2025 [19] NCT04992546	Atuzabrutinib	AD	IIa	39	Topical atuzabrutinib 5% gel BID versus vehicle in a split-lesion period, followed by open-label treatment	Safety and tolerability (primary); PK (secondary); exploratory lesion and pruritus measures showed no benefit over vehicle
Kircik 2025 [18] NCT05018806	Rilzabrutinib	AD	II	124	Placebo; rilzabrutinib 400 mg BID or 400 mg three times daily	Percentage change in EASI at week 16; not met for either dose (*p* = 0.62 and *p* = 0.67 versus placebo)
Yamagami 2021 [36]	Tirabrutinib	Pemphigus (vulgaris, foliaceus, and vegetans)	II	16	80 mg QD for 52 weeks with glucocorticoids	Complete remission at week 24; 3/16 (18.8%) in a single-arm study
Murrell 2021 [16] BELIEVE; NCT02704429	Rilzabrutinib	Pemphigus vulgaris	II	27	400–600 mg BID with oral glucocorticoids equivalent to a 0–0.5 mg/kg range of prednisolone	Control of disease activity within 4 weeks on zero-to-low-dose glucocorticoids and safety; achieved by 14/27 (52%)
Murrell 2024 [17] PEGASUS; NCT03762265	Rilzabrutinib	Pemphigus vulgaris or foliaceus	III	131 randomized	Rilzabrutinib 400 mg BID plus standardized glucocorticoid taper; placebo plus standardized taper	Complete remission in the 29–37 week range with corticosteroid dose ≤10 mg/day; not met in the PV primary analysis: 13/54 (24%) vs. 10/55 (18%); *p* = 0.45
Gudjonsson 2024 [20] NCT03827798	Remibrutinib	HS	II	77	Placebo; 25 mg BID; 100 mg BID	Simplified HiSCR50 at week 16; 72.7% (25 mg BID), 48.5% (100 mg BID), and 34.7% (pooled placebo; *n* = 49)
Kimball 2025 [42] NCT03827798	Remibrutinib	HS	II	77 (same cohort)	Placebo; 25 mg BID; 100 mg BID	Exploratory week-16 pain, itch, and DLQI outcomes; descriptive, as-observed analysis of the same platform-study cohort

Note: studies are grouped by disease and summarize study phase, sample size, treatment regimen, and the principal reported efficacy or safety outcome. “Main endpoint and result” reports the prespecified primary outcome when available; phase I and long-term extension studies are identified by their primary safety and tolerability objectives. The psoriasis studies cited here are preclinical [21,22,23]. The two HS reports describe the same remibrutinib cohort (33 patients per active arm and 11 concurrent placebo recipients); pooled placebo data from 49 patients were used for the main efficacy comparison and for pain/DLQI analyses, whereas the PRO itch analysis used concurrent placebo [20,42]. DLQI, Dermatology Life Quality Index; PV, pemphigus vulgaris; AD, atopic dermatitis; BID, twice daily; BTK, Bruton tyrosine kinase; CSU, chronic spontaneous urticaria; EASI, Eczema Area and Severity Index; HiSCR, Hidradenitis Suppurativa Clinical Response; HS, hidradenitis suppurativa; ISS7, weekly Itch Severity Score; MAD, multiple ascending dose; PD, pharmacodynamics; PK, pharmacokinetics; QD, once daily; SAD, single ascending dose; UAS7, weekly Urticaria Activity Score.

## 4. Discussion

Figure 3 summarizes candidate BTK-centered cellular pathways across immune-mediated skin diseases. The schematic provides mechanistic rationale but does not indicate equivalent clinical evidence across disorders. Current evidence is strongest for CSU, inconclusive in pemphigus and HS, negative for the rilzabrutinib primary AD efficacy endpoint with no exploratory benefit from topical atuzabrutinib, and preclinical in psoriasis.

From a clinical-development perspective, the evidence is most advanced for CSU. Remibrutinib significantly reduced UAS7 in two phase III trials, establishing short-term symptomatic efficacy in antihistamine-refractory CSU [15]. These trials do not establish elimination of autoimmunity or disease modification after withdrawal. Pemphigus presents a contrasting pattern: early uncontrolled studies showed clinical and glucocorticoid-sparing signals, whereas PEGASUS did not demonstrate a significant benefit for its primary remission endpoint [16,17]. The phase III result may indicate that BTK-dependent effects were insufficient to produce the expected clinical benefit, rather than merely that the population was heterogeneous or the endpoint was mistimed.

Endpoint timing remains a legitimate design consideration, but it cannot be used post hoc to discount a negative primary endpoint. Future pemphigus trials should retain clinically meaningful remission outcomes while separately measuring time to disease control, cumulative glucocorticoid exposure, PDAI, DSG1/DSG3 antibody titers, BTK target engagement, and relapse after withdrawal. Such measurements could determine whether biological target engagement occurs without adequate clinical benefit, but they should complement rather than replace prespecified clinical endpoints.

Evidence in AD, psoriasis, and HS studies further emphasizes that BTK is one node within larger inflammatory networks. The exploratory continuous-itch improvement reported with rilzabrutinib does not establish which cellular pathways mediated the change, and the primary EASI endpoint was not met [18]. The psoriasis intervention studies reviewed here are preclinical and involve dendritic cells, neutrophils, and γδ T-cell-associated responses [21,23]. HS provides an intermediate case: preliminary clinical activity and increased lesional BTK or STAT-family expression warrant further investigation, but expression alone does not establish pathway dependence [20,40]. Across these diseases, the question is whether BTK inhibition can produce a clinically meaningful benefit in the relevant population.

No biomarker discussed in this review has been prospectively validated to predict clinical response to a BTKi in an immune-mediated skin disease. Autoantibodies, complement, basophil or eosinophil counts, mast-cell activation measures, tissue BTK/SYK/FCGR/STAT3 expression, transcriptional signatures, and symptom burden are therefore mechanistically motivated candidates rather than clinically qualified predictive biomarkers. Short-term biomarker changes reported in CSU trials [29,30] are pharmacodynamic observations and do not by themselves show that a baseline marker selects responders. A testable prospective framework would prespecify one BTKi and collect disease-specific baseline measures, including autoantibody status, circulating immune-cell subsets, tissue pathway activity, disease severity, symptom burden, and prior treatment. The same study should measure early target engagement and biomarker change longitudinally and relate them to later outcomes: UAS7 or UCT in CSU; PDAI, complete remission, cumulative corticosteroid dose, and DSG titers in pemphigus; EASI-75 and PP-NRS in AD; and HiSCR with pain and itch outcomes in HS cases. Predictive value would require a prespecified treatment-by-biomarker interaction and validation in an independent cohort, not merely correlation with disease severity.

Combination or sequential treatment remains speculative. Pathway complementarity with anti-IgE, IL-4/13, JAK, IL-23, or IL-17 inhibition is a mechanistic hypothesis, not evidence that combined treatment will improve outcomes. Any prospective combination trial should first establish an additive clinical objective and should prespecify monitoring for infection, bleeding, cardiovascular events, drug interactions, and cumulative immunomodulation. Until such data are available, BTKi combinations should not be presented as an emerging clinical strategy.

Advances in delivery and molecular design could alter the efficacy-safety window, but clinical support is limited. Topical PRN473 suppressed antibody-mediated inflammation with low systemic accumulation in animal models [37], whereas topical atuzabrutinib did not improve AD lesions or pruritus in patients [19]. BTK degraders have been investigated mainly in B-cell malignancies as a strategy to overcome inhibitor resistance; these data do not establish efficacy or improved safety in immune-mediated skin diseases [48]. Integrating BTK occupancy, skin pharmacokinetics, and longitudinal outcomes could establish dose-exposure–response relationships; these tools should not be described as companion diagnostics until predictive performance is prospectively validated.

Route of administration should be matched to both disease distribution and the intended cellular target. Systemic exposure may be necessary when circulating autoreactive B cells, widespread mast-cell activation, or immune complexes sustain disease across multiple sites. Local delivery may be more appropriate when lesions are limited and the therapeutic aim is to suppress signaling within skin while minimizing systemic effects. The negative topical atuzabrutinib study on AD and the positive preclinical signal with PRN473 in immune-mediated skin models illustrate why local exposure alone is not sufficient. Future clinical studies of locally delivered BTK inhibitors will need to demonstrate adequate penetration, target occupancy, and a relationship between local pharmacology and clinically meaningful change.

Additional BTKis are being studied in immune-mediated disease. Newer molecules such as zanubrutinib have human pharmacokinetic and pharmacodynamic data [49], although this does not establish efficacy in immune-mediated skin disease. BTK-targeted positron emission tomography probes and other skin-directed imaging technologies may help quantify target engagement and optimize dose selection, but their ability to identify treatment-responsive skin lesions remains to be validated.

Safety should be interpreted by agent, selectivity, dose, exposure duration, population, and treatment setting rather than as a class-wide conclusion. In the eight-week fenebrutinib CSU trial, asymptomatic reversible grade 2–3 transaminase elevations occurred in the 150 mg once-daily and 200 mg twice-daily groups [29]. In the REMIX trials, overall and serious adverse-event rates were similar to placebo through the controlled period, but petechiae were more frequent with remibrutinib (3.8% vs. 0.3%) [15]. Rilzabrutinib AD treatment lasted 16 weeks and was associated mainly with mild events, including nausea and diarrhea [18], whereas the tirabrutinib pemphigus evidence derives from only 16 patients in an uncontrolled study [36]. These findings cannot establish long-term risks of serious infection, bleeding, cardiovascular or rhythm events, or malignancy across agents. Combination safety is entirely unestablished. Longer controlled follow-up, exposure-adjusted event reporting, withdrawal data, and real-world surveillance are required, particularly for chronic treatment and combined immunomodulation.

## 5. Conclusions

BTK links signaling in B cells and Fc-receptor-bearing effector cells, but the clinical relevance of this mechanism differs substantially among immune-mediated skin diseases. The available evidence supports the following disease-specific conclusions:

1. In individuals with CSU, phase III trials establish that remibrutinib improves wheals and itch in the 12–24-week range in adults who remain symptomatic despite H1 antihistamines. These data support symptomatic efficacy, not the elimination of autoantibody-producing plasma cells or durable disease modification after withdrawal.
2. In individuals with pemphigus, mechanistic rationale and uncontrolled early clinical signals have not been validated by a positive phase III primary endpoint. Clinical remission, glucocorticoid reduction, and antibody-titer changes remain distinct outcomes, and the overall clinical value of BTK inhibition is uncertain.
3. In individuals with HS, the randomized phase II signal is preliminary and the non-monotonic dose pattern requires confirmation. In individuals with AD, rilzabrutinib did not meet its primary EASI endpoint, whereas topical atuzabrutinib showed no benefit in exploratory efficacy assessments; the rilzabrutinib continuous-itch signal remains exploratory. In individuals with psoriasis, evidence is preclinical and does not support clinical treatment positioning.

Future research should use adequately powered, disease-specific prospective trials to validate predictive biomarkers, separate clinical from pharmacodynamic outcomes, define dose–response, and characterize long-term agent-specific safety. Combination regimens, topical formulations, and BTK degraders should remain investigational until their efficacy and safety are demonstrated directly.

## Data Availability

No new data were created or analyzed in this study. Data sharing is not applicable to this article.
